# Food stimuli decrease activation in regions of the prefrontal cortex related to executive function: an fNIRS study

**DOI:** 10.1007/s40519-023-01623-7

**Published:** 2023-11-20

**Authors:** Chen Cheng, Yong Yang

**Affiliations:** 1 Institute for Brain Sciences Research, Tennis College, Wuhan Sports University, Wuhan, 430079 China; 2 Graduate institute of athletics and coaching Science, National Taiwan Sport university, Taoyuan, Taiwan; 3 Present Address: Department of NeuroCognition/Imaging, School of Physical Education and Sport, Chaohu University, No. 1 Bantang Road, Chaohu City, Hefei City, 238000 Anhui Province China

**Keywords:** Food stimuli, Obesity, Executive function, Prefrontal cortex, fNIRS

## Abstract

**Purpose:**

Overweight/obese individuals show impairments in executive functions such as inhibitory control. However, the neural mechanisms underlying these disturbances—and specifically, whether or not they involve altered activation of the specific prefrontal cortex regions—are not yet fully understood.

**Methods:**

The motivational dimensional model of affect suggests that high approach-motivated positive affect (e.g., desire) may impair executive function. In the present study, we investigated individual differences in neural responses to videos of food stimuli, and examined brain activity during a cognitive task in an approach-motivated positive state using functional near-infrared spectroscopy (fNIRS). In Experiment 1, in 16 healthy young adults, we tested whether prefrontal cortex activation differed during a food video clip versus a neutral video clip. Then, after viewing each video clip, we tested for differences in executive function performance and prefrontal cortex activation during a Stroop task. Experiment 2 was the same, except that we compared 20 overweight/obese with 20 healthy young adults, and it incorporated only the food video clip.

**Results and conclusions:**

The results of both experiments indicated that food stimuli decrease activation in regions of the prefrontal cortex related to executive function. This study also suggests that overweight/obese might consciously suppress their responses to a desired stimulus, yet here it seems that effect was less pronounced than in healthy controls.

**Level of evidence:**

Level II, Cohort Studies.

## Introduction

Executive functions are higher-level cognitive processes that exert a top-down control of attention and unwanted affect, suppress ruminative thoughts and support the ability to switch between different behavioral cognitive and emotional strategies subserving the same or multiple goals [1]. Thus, impairment of executive functions may have serious consequences both for guiding behavior towards a goal and for emotional regulation.

Obese individuals often show deficits in executive functions compared to normal-weight participants [2]. There is research have shown that, according to a time-course analysis of the proportion of gaze to images of food and images of high-calorie food identified differences in attention processing between the obese group and control group, and the obese group had higher Stroop interference and track-making test b scores than the control group [3]. Indeed, in their meta-analysis, Yang et al. (2018) [4] found that obese participants showed broad impairments of executive function in a variety of tasks, including those primarily utilizing inhibition, cognitive flexibility, working memory, decision-making, verbal fluency, and planning; overweight participants showed significant impairments only in inhibition and working memory to those of normal-weight controls.

Obesity has been linked to structural and functional brain abnormalities, particularly in prefrontal cortical areas [5, 6]. Overweight/obese individuals with a greater hemodynamic response in the left ventrolataral prefrontal cortex (l-VLPFC) and bilateral dorsolateral prefrontal cortex (DLPFC) due to the Stroop effect lost more weight during a short-term fitness intervention than participants with lower levels of activation of these brain regions [7]. These findings suggest that prefrontal cortex activity related to executive function is associated with obesity.

According to the motivational dimensional model of affect [8], states high in approach motivation can occur in response to appetitive cues present in the environment [9], and it leads to overall narrowing of cognitive resources, so it may in turn impair executive function. Thus, repeated exposure to cues related to high-calorie foods may decrease activation in executive-function-related brain regions in some individuals, increasing craving and consumption of these foods and therefore contributing to unhealthy weight gain.

Recent studies have shown that in eating disorders deficits at the body level (interoception) carry with them deficits at the cognitive level, impacting the processing of the self [10]. This also occurs in healthy subjects as the interoceptive layer is embedded within the broader neural activation of the mental-cognitive layer [11]. Interoception is an ability to sense the physiological condition of the body [12]. Early clinical descriptions of Eating Disorder highlighted “impairments in the perceived accuracy or cognitive interpretation of stimuli from the body [13].” This difficulty in perceiving signals from the body and recognizing and interpreting emotional states has been established as some of the core psychopathological factors of eating disorder [14–16].

Different from existing research, in the present study, we try to investigate individual differences in neural responses to videos of food stimuli and examined brain activity during a cognitive task in an approach-motivated positive state using functional near-infrared spectroscopy (fNIRS) for understood the neural mechanisms underlying these disturbances and specifically, whether or not they involve altered activation of the specific prefrontal cortex regions.

To investigate the effect of desire for high-calorie foods on executive-function-related prefrontal cortex activity, the present study sought to characterize the neural response to food stimuli and examine brain activity during a cognitive task (Stroop task) in an approach-motivated positive state (desire) using functional near-infrared spectroscopy (fNIRS). Stroop task is widely used in experimental and clinical settings to measure executive functions localized in the prefrontal cortex [17]. fNIRS examines hemodynamic and neural activation during various cognitive tasks by measuring concentration changes in oxy-, deoxy-, and total-hemoglobin (Hb). fNIRS enables the analysis of cerebral activity with comparable spatial and temporal precision to other neuroimaging techniques [18, 19]. Moreover, fNIRS does not require large, immobile equipment that would restrict it to particular testing environments. Some studies have also used fNIRS to observe PFC activity during the Stroop task [20, 21]. Importantly, obese participants are also more likely to agree to fNIRS than other neuroimaging techniques [22].

In our study, we used 99-s-long video clips to induce particular emotions or desire in our participants. Although most studies of obese participants have been conducted using static pictures of appetizing foods, recent work in adolescents used video commercials of food instead [23]. Movie clip has several advantages over other methods [24], first, it has been widely observed that film excerpts can induce strong subjective and physiological changes [25, 26]. Secondly, the dynamic nature of the film set provides an optimal artificial model of reality, without the ethical and practical problems of real-life technology. Third, it is one of the easiest techniques to implement in a lab. Fourth, it appears to be the most powerful technique for eliciting emotions in the lab [27]. Film clips have also been widely used and accepted as effective stimuli in the field of emotion research [24, 28, 29]. In contrast to static pictures, films are dynamic and thus thought to have an increased degree of realism [30]. Some studies have concluded that films effectively induce emotions, especially positive emotions [27, 31].

The purpose of this study from the perspective of motivational dimension model of emotion, whether food stimulation damage's executive function is explored. To our knowledge, no fNIRS studies have observed the brain response to video clips of food and neutral objects and examined brain activity during the Stroop task in a high approach-motivated positive state (desire). We conducted two experiments. In Experiment 1, in 16 healthy young adults, we tested whether prefrontal cortex activation differed during a video clip depicting food versus a neutral video clip. Then, after viewing each video clip, we tested for differences in executive function performance and prefrontal cortex activation during a Stroop task. The objective of Experiment 1 was to investigate whether food stimuli can degrade performance on an executive function task. Experiment 2 was the same, except that we compared 20 overweight/obese with 20 further healthy young adults, and it incorporated only the food video clip. The objective of Experiment 2 was to investigate whether food stimuli have the different effect on the executive function of overweight/obese and healthy controls (HC). Through these two studies combined, we achieved a thorough investigation of the effects of desired state on executive-function-related prefrontal cortex areas.

## Experiment 1

### Methods

#### Participants

16 healthy young adults (9 male and 7 female, 18 to 26 years old) with a mean body mass index (BMI) of 20.8 ± 2.6 kg/m^2^ completed the experiment. The demographic characteristics of the participants are shown in Table 1.

**Table 1:** Characteristics of participants

	Male	Female	*p* value
Number of participants	9	7	
Age (years)	19.00 ± 0.71	19.00 ± 0.82	1.000
BMI (kg/m^2^)	21.02 ± 1.51	19.70 ± 1.78	0.131
Neutral condition			
Pleasing	2.00 ± 1.12	2.57 ± 1.99	0.477
Arousing	5.67 ± 1.00	5.29 ± 0.76	0.417
Desirable	3.67 ± 1.58	3.00 ± 1.92	0.458
Food stimuli condition			
Pleasing	7.11 ± 2.47	8.00 ± 0.82	0.380
Arousing	8.11 ± 0.78	8.43 ± 0.54	0.375
Desirable	7.00 ± 1.58	7.29 ± 0.49	0.654

Following a comprehensive description of the methods and procedures involved, written informed consent was obtained. Eligibility criteria for study participation were: (1) native Chinese speaker, (2) right-handed, (3) normal or corrected-to-normal vision [eyeglasses or contact lenses], (4) normal color vision, and (5) no self-reported history of any mental disorder. The Ethics Committee of Wuhan Sports University approved this research.

### Procedure

For all participants, the experiment was scheduled in the afternoon. Each participant was requested to consume a regular lunch 1 to 1.5 h before he or she arrived at the laboratory. Subjective hunger ratings and affect were assessed before the start of the experiment. Additionally, the participant’s weight and height were measured.

We showed to all participants a neutral video clip and a food video clip in an ABBA protocol. The neutral video clip that showed repairing computer was obtained from Native Chinese Affective Video System, and the food video clip that showed cooking high-calorie and palatable foods (e.g., chocolate, cream, steak, chicken leg, hamburger, etc.) were obtained from open-access videos on the World Wide Web. All video clips lasted 99 s and have sound. After viewing each video clip, participants completed Stroop task, which assesses executive function. Finally, participants rated how desired the video clip made them feel (1 = no emotion, 9 = strongest feeling). Participants also rated how pleasing 1 (very unpleasing) to 9 (very pleasing) and arousing 1 (calm) to 9 (exciting) was, using the Self-Assessment Manikin.

### fNIRS Stroop paradigm

**Fig. 1:**
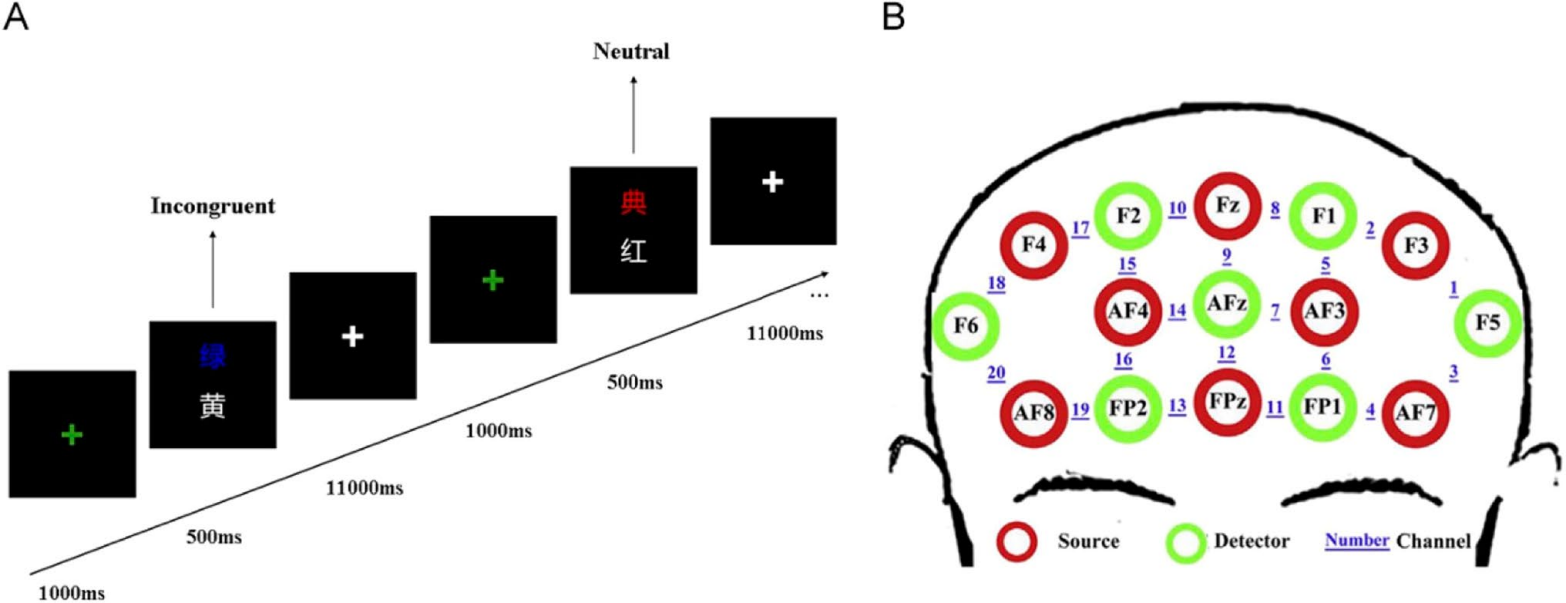
An illustration of the Stroop task and spatial arrangement of the fNIRS probes. **A** The experimental design of the Stroop task and an example of the neutral and incongruent conditions. 红 means “red”, 黄 means “yellow”, 绿 means “green”, and 典 means “ceremony”. The participants were instructed to decide whether the color of the upper character matched the meaning of the lower one. **B** Spatial arrangement of the fNIRS probes. The red circles indicate the 8 sources, the green circles indicate the 7 detectors, and the blue numbers (1–20) indicate the fNIRS channel numbers. The optical sources and the detector probes were placed according to the International 10–20 standard positions

The Stroop task used for this study was an event-related version designed using E-prime 2.0 (Psychology Software Tools, Pittsburgh, PA, USA). The experimental paradigm is based on researches of Zhang et al. (2013) [21], Zhai et al. (2009) [20] (see Fig. 1A). The stimuli consisted of two Chinese characters, and participants were instructed to decide whether the color of the upper one matched the meaning of the lower one. If the answer was “Yes”, participants pressed the F button with the index finger of their left hand, whereas if the answer was “No”, they pressed the J button with the index finger of their right hand. There were two kinds of stimuli conditions: neutral and incongruent. In neutral stimuli, the upper Chinese character was a non-color-meaning word (晏, 贯, 典, 俞, meaning “comfort”, “through”, “ceremony”, “a surname”) or presented in green, yellow, red, blue, and the lower Chinese character was a color-meaning word (红, 黄, 蓝, 绿, meaning “red”, “yellow”, “blue”, “green”) presented in white. In incongruent stimuli, the upper Chinese character was a color-meaning word printed in a disparate color. In order to prevent participants from ignoring the upper Chinese character, we asked them to attend to the upper Chinese character first before making the judgment. For each stimuli condition, the numbers of “Yes” trials and “No” trials were equal, and the two kinds of trials were semi-randomly mixed in order to avoid the consecutive appearance of more than three trials in the same category.

### fNIRS data recording

A multi-channel continuous wave fNIRS instrument (NIRScout, NIRx Medical Technologies LLC, USA) was used to monitor hemodynamic activity. The fNIRS probe consisted of 8 sources (760 and 850 nm) and 7 detectors, which covered the frontal area (see Fig. 1B). The distance between the emitter and the detector was 3 cm. The probe arrangement was related to the 10/20 EEG positions, and fine adjustments were made to ensure that the distance between the emitter and the detector was 3 cm. Okamoto and Tsuzuki confirmed the relationship between the location of the fNIRS channel and specific brain regions.

### fNIRS data processing and analysis

Optical data were converted into Hb signals in an arbitrary unit using the modified Beer–Lambert law. The sampling rate was 7.8 Hz. The Hb data were bandpass filtered between 0.01 Hz and 0.3 Hz to remove baseline drift and physiological noise (e.g., heartbeats). Each participant’s hemodynamic data were averaged across the 24 trials for the congruent and incongruent conditions. According to previous studies, deoxy-Hb signals have a worse signal-to-noise ratio than oxy-Hb signals, and deoxy-Hb signals have been shown to reflect no significant Stroop effect in some fNIRS studies [32, 33]. Therefore, as in other studies, oxy-Hb signals were employed to estimate regional brain activity. Previous findings also showed that oxy-Hb was the most sensitive marker for task-related hemodynamic changes [17].

We recorded the mean concentration change of the oxy-Hb signals across the different conditions and channels. These indicators revealed the hemodynamic response for the experimental control. In this way, we were able to compare the brain activities of two groups. We used SPSS 17.0 software (SPSS Inc., Chicago, IL, USA) to analyze the hemodynamic data. Significance was analyzed using t-test.

### Behavioral data analysis

The mean number of trials with no responses averaged across participants was less than 1 for both stimuli conditions, and these missed trials were not used for the behavioral data analysis. For response time analysis, only trials with response time lying within three standard deviations of the mean value were used.

## Results

The food video clip was rated as more pleasing, arousing, and desirable than the neutral video clip by the HC (t(15) = 11.000, *p* < 0.01; t(15) = 8.474, *p* < 0.01; t(15) = 8.927, *p* < 0.01). These self-reported responses to the video clips suggest that the food stimuli may have induced desire to high-calorie foods.

### Stroop task data

**Fig. 2:**
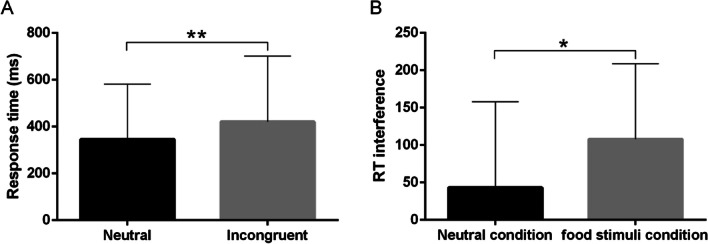
Response time and reaction time interference for the incongruent and neutral conditions during the fNIRS exam. **A** A longer response time was observed for the incongruent condition than for the neutral condition. **B** A higher reaction time interference was observed for the food stimuli condition than for the neutral condition. Paired t-test. **p < 0.01. *p < 0.05

We analyzed the response times associated with the neutral and incongruent Stroop task conditions. The results suggested significantly longer response times for the incongruent condition than for the neutral condition (*p* < 0.01, see Fig. 2A), showed significant Stroop effects.

Regarding reaction time interference (i.e., the incongruent minus the neutral trials; see Fig. 2B), there was also a significant difference (t(15) = − 2.907, *p* < 0.05) between the neutral condition (43.17 ± 114.74 ms) and the food stimuli condition (108.10 ± 100.63 ms).

### fNIRS data

**Fig. 3:**
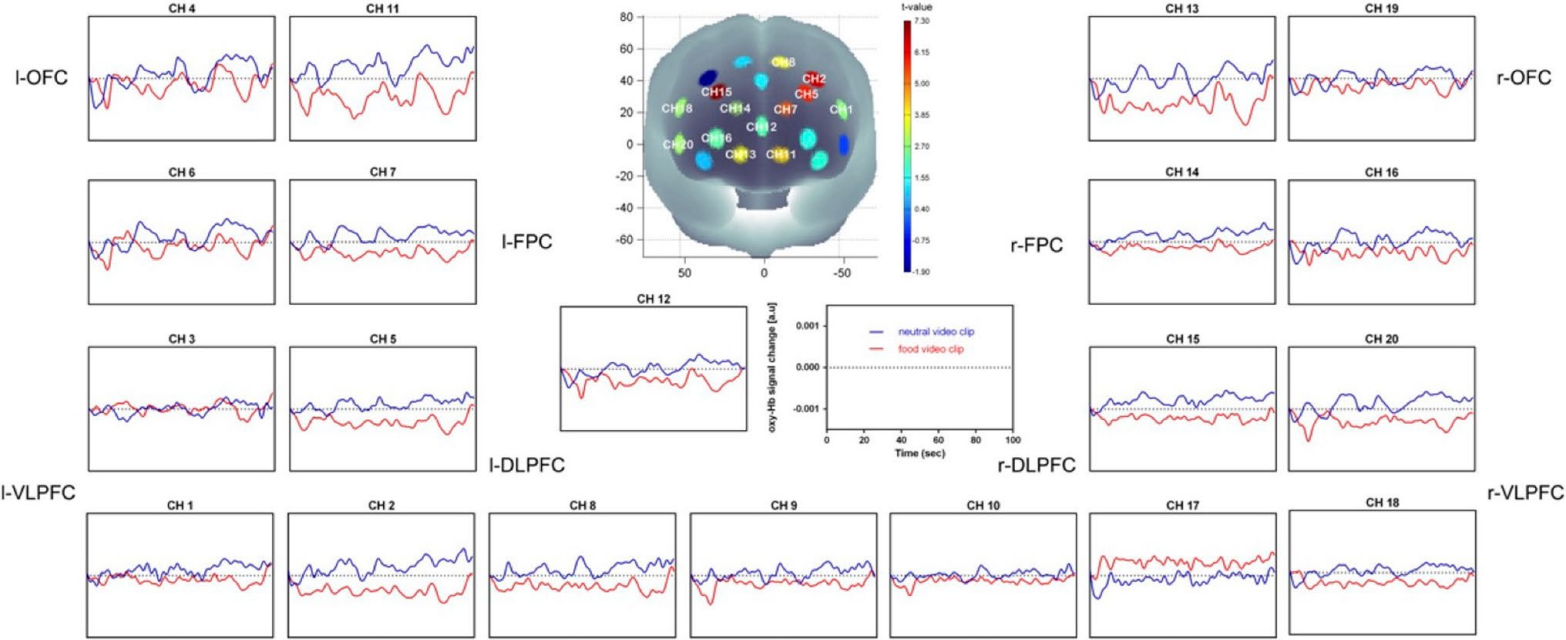
The time courses of the oxy-Hb signal changes in the 20 channels during neutral and food stimuli video clips. Higher activation in the VLPFC (CH 1, CH 18, CH 20), DLPFC (CH 2, CH 5, CH 8, CH 15), FPC (CH 7, CH 12, CH 14, CH 16) and OFC (CH 11, CH 13) was observed during the viewing of the neutral video clip than during the food stimuli video clip

We compared the time course of the oxy-Hb concentration changes in areas of the PFC while participants viewed the neutral video clip versus the food video clip (see Fig. 3). In the VLPFC (CH 1: t(15) = 2.724, *p* < 0.05; CH 18: t(15) = 2.899, *p* < 0.05; CH 20: t(15) = 2.942, *p* < 0.05), DLPFC (CH 2: t(15) = 6.620, *p* < 0.01; CH 5: t(15) = 5.890, *p* < 0.01; CH 8: t(15) = 3.873, *p* < 0.01; CH 15: t(15) = 7.270, *p* < 0.01), frontopolar cortex (FPC, CH 7: t(15) = 5.373, *p* < 0.01; CH 12: t(15) = 2.251, *p* < 0.05; CH 14: t(15) = 3.031, *p* < 0.01; CH 16: t(15) = 2.339, *p* < 0.05), and OFC (CH 11: t(15) = 4.220, *p* < 0.01; CH 13: t(15) = 3.844, *p* < 0.01), we found that the participants had higher activation during the neutral clip than during the food clip.

**Fig. 4:**
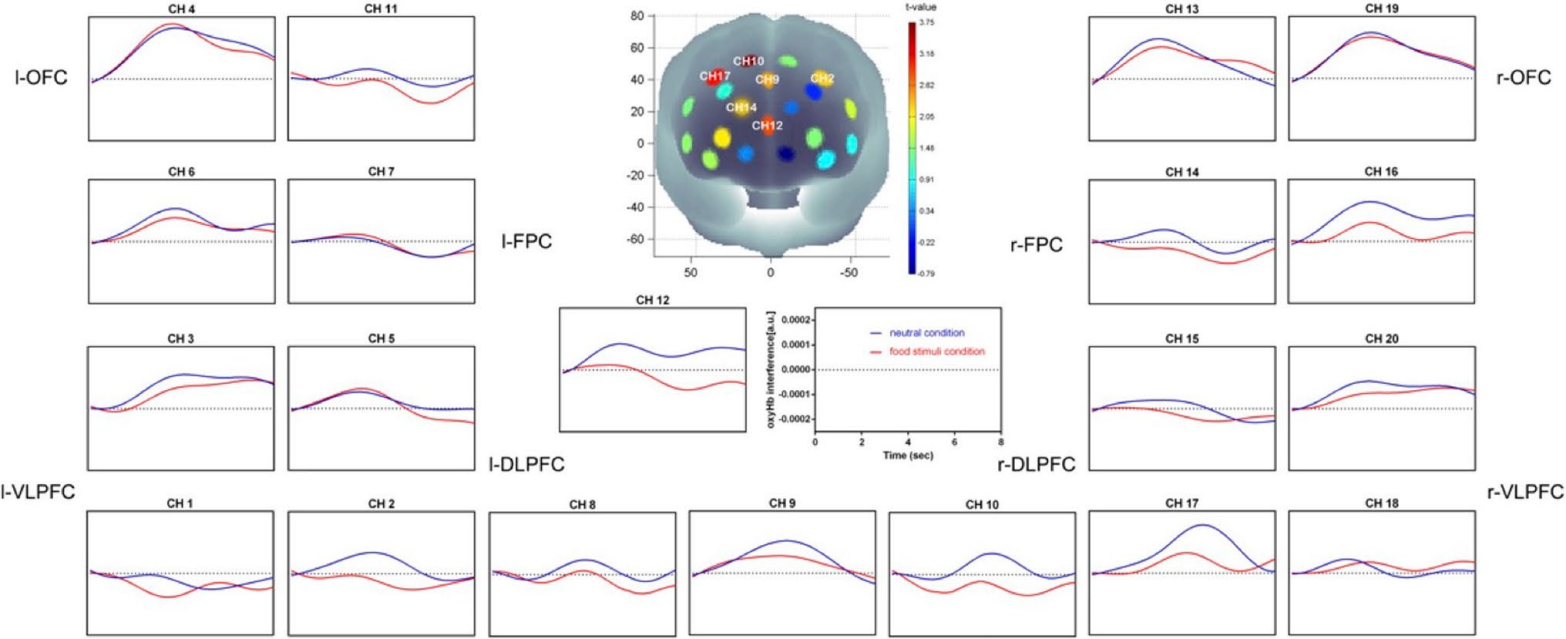
The time courses of the oxy-Hb responses in the 20 channels in the neutral and the food stimuli condition of the Stroop task. Higher oxy-Hb interference in the DLPFC (CH 2, CH 9, CH 10, CH 17) and r-FPC (CH 12, CH 14) was observed in the neutral condition than in the food stimuli condition

There were significant Stroop effects in CH 3, CH 8, CH 9, CH 12, CH 13, CH 14, CH 16, and CH 20 (*p* < 0.05), with a higher oxy-Hb response for the incongruent task than for the neutral task. Significantly higher oxy-Hb interference (i.e., the incongruent minus the neutral trials) was observed in the neutral condition than the food stimuli condition for the DLPFC (CH 2: t(15) = 2.288, *p* < 0.05; CH 9: t(15) = 2.437, *p* < 0.05; CH 10: t(15) = 3.750, *p* < 0.01; CH 17: t(15) = 3.121, *p* < 0.01) and the right frontopolar cortex (CH 12: t(15) = 2.857, *p* < 0.05; CH 14: t(15) = 2.247, *p* < 0.05) (see Fig. 4).

## Discussion

During the video clips, we found higher activation during the neutral clip than during the food clip in the VLPFC, DLPFC, frontopolar cortex (FPC), and OFC of HC, possibly suggesting that the HC invested more cognitive resources in neutral stimuli than in food stimuli. Our results are inconsistent with other reports. An fMRI study found that viewing photographs of calorie-dense food compared to nonfood objects resulted in significantly increased activation in the l-DLPFC and l-OFC [34]. One possible explanation for this discrepancy is that our study used video clips rather than photos to induce emotions or desire. The induction of emotions by video clips is more complex than the elicitation of emotions by pictures, since it comprises some perceptual elements (i.e., lighting, camera-angle or the use of colors) [35]. The activation of the OFC may reflect the subjects focusing their attention on neutral cues. An fMRI study found that BMI was negatively correlated with activation in the OFC as participants focused their attention on unappetizing food images [36]. It is possible that the HC invested more attentional resources when viewing the neutral clip than when viewing the food clip. The VLPFC forms an important part of the circuitry in which associations between visual cues and the actions or choices they specify are formed and is thought to play a role in selecting the correct course of action out of multiple behavioral choices [37]. The neutral video clip showed the process of repairing a computer. Thus, compared to the food video clip, which showed only high-calorie and palatable food, HC might have invested more cognitive effort in thinking about how to repair a computer when viewing the neutral video clip. The changes in the resting brain activity in the DLPFC tend to be more pronounced in individuals who experience less pleasure from food. Most of the previous studies on the topic have demonstrated that the DLPFC plays a critical role in self-control of food consumption [38, 39]. Cognitive self-control of the desire for food might affect the expression of positive emotion toward eating, such as pleasure in food consumption, via this brain mechanism. Thus, the lower the activation of the DLPFC in HC viewing the food clip, the happier they presumably felt when viewing the clip. The FPC is reportedly involved in memory retrieval [40–42] as well as memory encoding and recognition [41, 43, 44]. Thus, increased activation in the FPC during the neutral clip suggested that HC might have been memorizing how to repair a computer when viewing the clip. Some studies also suggest that updating emotional memory depends on the FPC/OFC [45]. Thus, our results suggest that HC might be absorbed in watching food instead of remembering food when viewing the food video clip. The motivational dimensional model of affect theory predicts that high approach-motivated positive affect, such as desire, should result in a narrowing of attention [46]. Thus, higher activation in the VLPFC, DLPFC, FPC, and OFC during the neutral clip suggested that more brain regions would be activated in HC when their breadth of attention was increased.

During the subsequent Stroop task, both reaction time interference and fNIRS data showed significant Stroop effects. Higher reaction time interference was observed in the food stimuli condition than in the neutral condition. In addition, higher oxy-Hb interference in the DLPFC and r-FPC was observed in the neutral condition than in the food stimuli condition. These Stroop effect findings are similar to those in previous neuroimaging studies that suggest that executive function is associated with activation of specific PFC regions, including the r-FPC and DLPFC [47–49]. As these areas have been shown to play a key role in response inhibition, one explanation for our observations is that the neural circuitry underlying inhibitory control may be recruited less after viewing the food clip than after viewing the neutral clip. The results of Experiment 1 revealed that food stimuli decrease activation in executive-function-related regions of the prefrontal cortex, suggesting that desire might lead to poor cognitive task performance and poor inhibitory control, traits that have been previously shown to contribute to unhealthy eating patterns and weight gain. Experimental results support the motivational dimensional model of affect theory, high approach-motivated positive affect may cause cognitive deficits.

## Experiment 2

The results of Experiment 1 revealed that a food stimulus decreased activation in executive-function-related regions of the prefrontal cortex in HC. In Experiment 2, we examined whether overweight/obese and HC have different performance and brain activation after viewing the same food stimulus as in Experiment 1, and during the Stroop task.

## Methods

### Participants

20 overweight/obese participants (11 male and 9 female, 18 to 24 years old) with a mean BMI of 29.46 ± 7.16 kg/m^2^ and 20 healthy participants (12 male and 8 female, 18 to 26 years old) with a mean BMI of 20.75 ± 1.35 kg/m^2^ completed the experiment. Overweight/obese participants were recruited from a private fitness and weight loss summer camp. HC were newly recruited and did not include the previous participants. Overweight/obese participants and HC did not show significant differences with respect to age and sex distribution. The demographic characteristics of the participants are shown in Table 2.

**Table 2:** Characteristics of participants

	Overweight/obese participants	Healthy controls	*p* value
Number of subjects	20	20	
Age (years)	20.30 ± 3.84	19.05 ± 0.94	0.166
Gender (female: male)	8:12	11:9	0.342
BMI (kg/m^2^)	29.46 ± 7.16	20.75 ± 1.35	0.000
Self-report			
Pleasing	7.85 ± 1.39	7.05 ± 1.57	0.096
Arousing	6.35 ± 1.69	5.50 ± 0.95	0.057
Desirable	6.05 ± 2.93	5.00 ± 2.87	0.259

Following a comprehensive description of the methods and procedures involved, written informed consent was obtained. Eligibility criteria for study participation were: (1) native Chinese speaker, (2) right-handed, (3) normal or corrected-to-normal vision [eyeglasses or contact lenses], (4) normal color vision, and (5) no self-reported history of any mental disorder. The Ethics Committee of Wuhan Sports University approved this research.

### Procedure

#### Experiment one

Watching neutral videos and complete STROOP task → Watch food videos and complete STROOP task (fNIRS monitoring throughout, the purpose of this experiment is to investigate whether food stimulation can reduce the executive function of normal-weight subjects, neutral stimulation as a control, the viewing order of the two videos is balanced between subjects).

#### Experiment two

Control group group: Watch neutral videos (watch only, no fNIRS) → Watch food videos and complete STROOP task (recording fNIRS).

Obese group: Watch neutral videos (watch only, no fNIRS) → Watch food videos and complete STROOP task (recording fNIRS).

For all participants, the experiment was scheduled in the afternoon. Each participant was requested to consume a regular lunch 1 to 1.5 h before he or she arrived at the laboratory. Subjective hunger ratings and affect were assessed before the start of the experiment. Additionally, the participant’s weight and height were measured.

All participants viewed the neutral video clip. This was to make overweight/obese participants the same starting state as HC. Next, participants viewed the food video clip. After viewing the food video clip, participants completed Stroop task. Finally, participants rated how desired the video clip made them feel (1 = no emotion, 9 = strongest feeling). Participants also rated how pleasing 1 (very unpleasing) to 9 (very pleasing) and arousing 1 (calm) to 9 (exciting) was, using the Self-Assessment Manikin.

### fNIRS Stroop paradigm

The same as Experiment 1.

### fNIRS data recording

The same as Experiment 1.

### fNIRS data processing and analysis

We recorded the mean concentration change of the oxy-Hb signals across the different participants, conditions and channels. These indicators revealed the hemodynamic response for the experimental control. In this way, we were able to compare the brain activities of two groups. We used SPSS 17.0 software (SPSS Inc., Chicago, IL, USA) to analyze the hemodynamic data. Significance was analyzed using t-test.

### Behavioral data analysis

The mean number of trials with no responses averaged across participants was less than 1 for both stimuli conditions, and these missed trials were not used for the behavioral data analysis. For response time analysis, only trials with response time lying within three standard deviations of the mean value were used.

## Results

Overweight/obese participants rated the food video clip as more pleasing, arousing and desirable than HC did; however, the differences between the two groups did not reach statistical significance (*p* > 0.05, see Table 2).

### Stroop task data

**Fig. 5:**
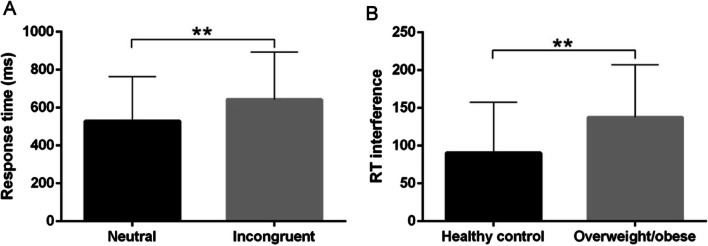
Response time and reaction time interference for the incongruent and neutral conditions during the fNIRS exam. **A** A longer response time was observed for the incongruent condition than for the neutral condition. Paired t-test. **B** A higher reaction time interference was observed for overweight/obese participants than for HC. Independent sample t-test. **p < 0.01

We analyzed the response times associated with the neutral and incongruent Stroop task conditions. The results suggested significantly longer response times for the incongruent condition than for the neutral condition (*p* < 0.01, see Fig. 5A), showed significant Stroop effects.

There was also a significant difference in reaction time interference (t(38) = 2.455, *p* < 0.01) between overweight/obese participants (137.61 ± 69.66 ms) and HC (90.27 ± 67.20 ms) (see Fig. 5B).

### fNIRS data

**Fig. 6:**
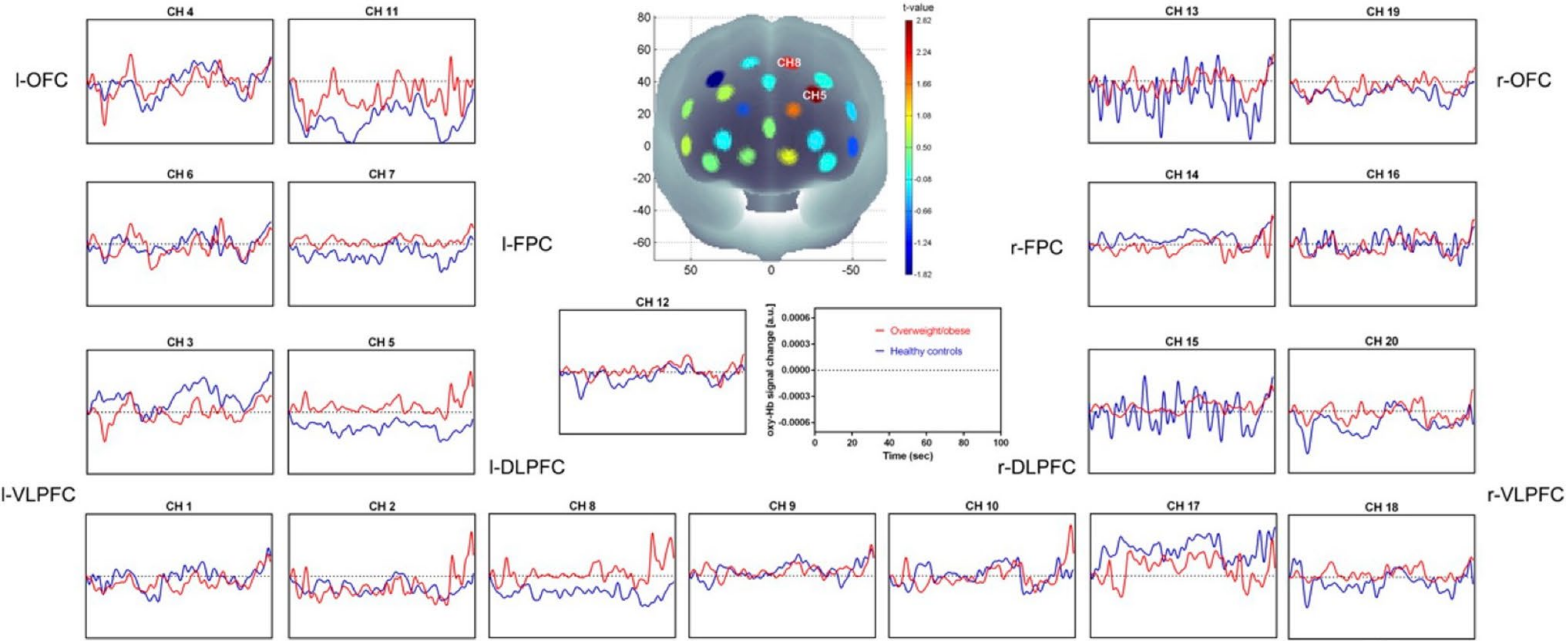
The time courses of the oxy-Hb signal changes in the 20 channels during the food stimuli video clip. Higher activation in the l-DLPFC (CH 5, CH 8) was observed during the viewing of the food stimuli video clip in overweight/obese participants than in HC

We compared the time course of the oxy-Hb concentration changes between overweight/obese participants and HC during the presentation of the food video clip (see Fig. 6). We found greater activation in the l-DLPFC (CH 5: t(38) = 2.824, *p* < 0.01; CH 8: t(38) = 2.213, *p* < 0.05), in overweight/obese participants than in HC.

**Fig. 7:**
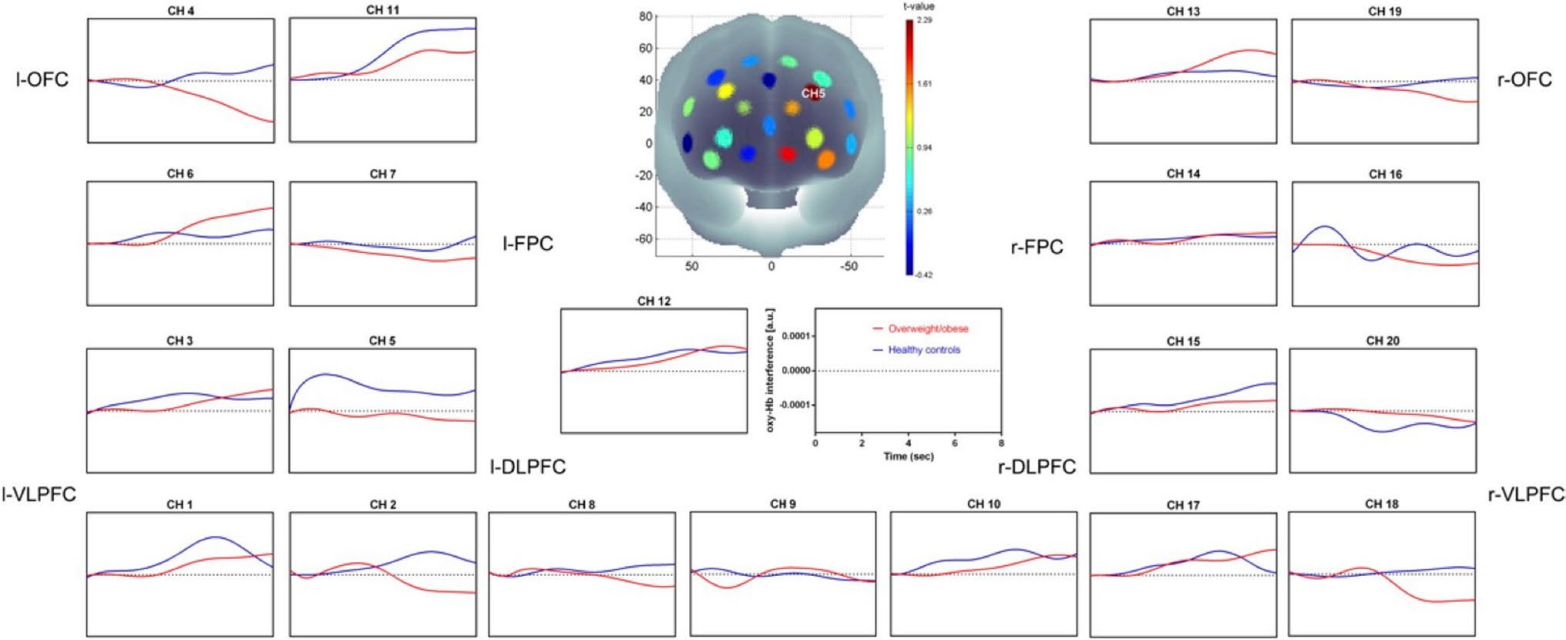
The time courses of the oxy-Hb response in the 20 channels during the Stroop task. Higher oxy-Hb interference in the l-DLPFC (CH 5) was observed in HC than in overweight/obese participants

There were significant Stroop effects in CH 2, CH 6, CH 9, CH 12, CH 16, and CH 17 (*p* < 0.05), with higher oxy-Hb response for the incongruent task than for the neutral task. Significantly higher oxy-Hb interference (i.e., the incongruent minus the neural trials) in the l-DLPFC was observed in HC than in overweight/obese participants (CH 5: t(38) = − 2.048, *p* < 0.05) (see Fig. 7).

## Discussion

During the food clip, we found higher activation in the l-DLPFC in overweight/obese participants than in HC. Our results are inconsistent with some reports. A meta-analysis of fMRI studies found that obese participants, in comparison to healthy-weight participants, had reduced activation in the l-DLPFC and left insular cortex in response to food images [50]. One explanation for this discrepancy could be that maintained attention, for example to a 99-s video clip, is associated with activation of the l-DLPFC [51]. An eye-tracking study showed that attention for food did not differ on any of the attentional bias measures between children with obesity and healthy-weight children, yet initial orientation bias to food was related to reduced weight loss after 6 months in children with obesity [52]. Thus, studies using food images might investigate initial attention bias to food, rather than maintained attention, as in our study. Indeed, considering different temporal components of attention is important because they are thought to reflect automatic (initial, shorter attention) versus controlled (prolonged attention) processes [53]. Research has suggested that these different temporal components of attention might differentially affect eating behavior and food cravings [54, 55]. It is thus possible that overweight/obese participants invested more attentional resources than HC in viewing the food video clip. In addition, an EEG study found that normal-weight participants had stronger l-DLPFC activation than obese participants when admitting a desire, while the same region was more strongly activated in obese than normal-weight participants when regulating a desire [56]. In our study, each participant was asked to consume a regular lunch 1 to 1.5 h before they arrived at the laboratory. Thus, participants might have regulated, rather than admitted, their desire because it was not time for dinner yet. Our fNIRS data suggest the possibility that it is more difficult for overweight/obese participants than HC to regulate their desire for food, and that the former invested more cognitive resources in regulating their desire for food. An fMRI study showed that obese children showed increased activation of the DLPFC in response to food pictures and high inhibitory control responses to food stimuli [57]. The authors explained that this inhibitory top-down control is exerted by the DLPFC, suppressing the activation of sub-cortical structures, diminishing their ability to detect the rewarding value of external cues, and to integrate internal body state information. Hence, because of their strong inhibitory control, obese children cannot be easily affected by external cues and have difficulty reconciling these cues with internal body needs. This results in early disorders of food intake regulation and eating behavior, consequently contributing to obesity [57]. These results could suggest that, in our study, overweight/obese participants reacted with high inhibitory control to food stimuli. In addition, the motivational dimensional model of affect theory holds that left prefrontal cortical regions are associated with local attention and approach-motivated affect, such as desire [58]. Thus, activation during the viewing of the food clip in the l-DLPFC of overweight/obese participants suggested increased approach-motivated positive affect and an increase in local attention.

During the subsequent Stroop task, both reaction time interference and fNIRS data showed significant Stroop effects. Higher reaction time interference was observed in overweight/obese participants than in HC. In addition, higher oxy-Hb interference in the l-DLPFC was observed in HC than in overweight/obese participants. These Stroop effect findings are similar to those in previous neuroimaging studies, which suggest that executive function is associated with activation of specific PFC regions, including the l-DLPFC [17, 33, 59, 60]. Numerous neuroimaging studies have suggested an association between obesity and deficits in activity in brain regions mediating executive function, such as the prefrontal cortex [61–63]. Experiment 2 revealed that overweight/obese participants had decreased performance on a test of executive function after viewing the food video clip compared to HC. One study, using a food Stroop task in which participants named the color of a colored food-related word or a colored neutral word [64], showed that children with obesity performed worse than healthy-weight children. This suggests that obese children found it more difficult than healthy children to suppress the processing of the meaning of food-related words, and might thus be more preoccupied with food. Relating these findings to our study, we hypothesize that overweight/obese participants used more cognitive resources in response to food stimuli, available cognitive resources were reduced during the subsequent Stroop task, making it difficult to compare the color of the upper Chinese character with the meaning of the lower one.

### General discussion

Experiment 1 showed that food stimuli decrease activation in regions of the prefrontal cortex related to executive function in Stroop tasks in HC. We found that brain activation was weaker in HC when viewing food stimuli than when viewing neutral stimuli. Therefore, their decreased activation in DLPFC and r-FPC in Stroop task may be a continuing adverse effect of food stimuli on the brain. That is, food stimuli reduced the level of brain activation, leaving a decreased amount of executive function for the subsequent Stroop tasks. Thus, HC had decreased performance on the Stroop task after viewing food stimuli. Experiment 2 showed that lower activation in regions of the prefrontal cortex related to executive function in Stroop tasks in overweight/obese participants. We found that higher oxy-Hb interference only in the l-DLPFC was observed in HC compared to the overweight/obese. However, we found higher activation in the l-DLPFC during the food video clip in overweight/obese participants than in HC. This finding suggests that overweight/obese participants need to invest more attention resources during the food video clip and more cognitive resources in regulating their desire, then they had decreased cognitive resources in the subsequent Stroop task than HC.

### Strength and limitation

Previous studies have already demonstrated that exposure to food cues plays a role in both the development and the persistence of obesity. In order to know how they contribute to obesity, further research into the neural basis of obesity should be done and provide knowledge about their relationship with top-down factors in obese adolescents [3]. Our research facilitates understanding the brain mechanisms of obesity by brain imaging technique. The results on this subject indicated that food stimuli decrease activation in regions of the prefrontal cortex related to executive function. It also suggests that overweight/obese might consciously suppress their responses to a desired stimulus. Furthermore, there is a need to reflect on the fact that the administration of these stimuli of this research implies the involvement of multiple sensory areas: movie clips are multimodal stimuli involving the visual and auditory systems. This may have important consequences in the neural activation that needs to be discussed. Furthermore, it may play a role in their conflicting results. This deficiency should be supplemented and modified in future studies.

## Conclusions

We conclude that food stimuli decrease activation in executive function-related regions of the prefrontal cortex. Our method of using visual food stimuli and an executive function task might be a tool to study the neural basis of obesity. Based on studies of the brain mechanisms of obesity, clinical workers can also apply some measures (e.g., transcranial magnetic stimulation intervention) to the executive-function-related regions of the prefrontal cortex in obese patients to help manage their food intake.

### What is already known on this subject?

Food cues and obesity: Existing research has robustly demonstrated that exposure to food cues, such as visual stimuli like advertisements, the smell of food, and food imagery, plays a pivotal role in both the development and persistence of obesity. These cues can stimulate appetite and lead to overeating, contributing to the accumulation of excess body weight over time.

Neural basis of obesity: Investigating the neural basis of obesity is critical for a comprehensive understanding of how food cues influence this complex condition. Advances in neuroimaging techniques, such as functional MRI (fMRI) and PET scans, have allowed researchers to identify specific brain regions and neural circuits associated with food craving, reward processing, and impulse control. Understanding the neural mechanisms involved in response to food cues can provide valuable insights into the biological underpinnings of obesity.

Top-down factors in obese adolescents: It is essential to explore how neural responses to food cues interact with top-down factors in obese adolescents. Top-down factors include cognitive and psychological processes like self-regulation, executive function, and emotional regulation. Research should aim to uncover how these top-down factors modulate the neural responses to food cues in obese adolescents, potentially shedding light on targeted interventions and therapies to address the obesity epidemic among young individuals.

### What does this study add?

Firstly, our research has demonstrated that exposure to food stimuli has a distinct impact on the brain. We have observed a decrease in activation within regions of the prefrontal cortex that are intimately associated with executive function. This indicates that when individuals, particularly those who are overweight or obese, are exposed to tempting food cues, their ability to engage in cognitive processes related to self-control, decision-making, and impulse regulation may become compromised. This discovery underscores the neurological basis for the vulnerability to overeating and the challenge of maintaining a healthy diet in environments saturated with food-related stimuli. Secondly, our research has shed light on a potentially conscious effort by overweight and obese individuals to suppress their responses to desired stimuli. This conscious suppression mechanism is a fascinating aspect of our findings. It suggests that individuals struggling with weight management may be aware of their heightened susceptibility to food cues and are actively attempting to counteract these impulses. This knowledge provides new insights into the psychological and cognitive strategies employed by individuals dealing with obesity and may serve as a basis for developing more effective intervention strategies and treatments.

## Data Availability

Data supporting the results of the study can be made available by emailing the first author or corresponding author.
